# The Hospital. Nursing Section

**Published:** 1905-02-18

**Authors:** 


### The Hospital.

### Nursing Section.

Contributions for this Section of "The Hospital" should be addressed to the Editor, "The Hospital," Nursing Section, 28 & 29 Southampton Street, Strand, London, W.C.

No. 960.—Vol. XXXVII. *SATURDAY, FEBRUARY* 18, 1905.

### Notes on News from the Nursing World.

### THE NURSES' CO-OPERATION.

The report and accounts of this Co-operation for the year ended December 31st, 1904, show that there are now 491 nurses on the general staff, and 23 asylum trained nurses who take mental cases only. It has been decided to add the nurses who joined the Co-operation during 1899 to those paying the decreased percentage of 5 per cent. commission, when 337 of the total staff of 491 nurses will have the advantage of these exceptional terms. After dividing £42,418 amongst the nurses, the commission on the fees, with interest on investments, yielded £2,859. This income of £2,859 sufficed to pay all the working expenses of the co-operation, together with the charge in respect to the Home and Vandyck Mansions, and left a balance in hand on the year's working of £855. The auditors, Messrs, Craggs, Turketine and Co., are to be congratulated on the clear and detailed way in which the accounts are rendered. We also congratulate Miss Baker on the success of her management of the Nurses' Home and Club, which has resulted in making this branch of the work nearly self-supporting, as there is only a small debit balance of some £16. In view of the losses from the club in previous years this result entitles Miss Baker to be regarded as the right woman in the right place.

### ULTIMATUM OF ASYLUM NURSES

A threatened strike of 26 nurses at Ryhope Asylum, Sunderland, has been averted. According to the regulations the night nurses are off duty at 6 A.M., and the day nurses are required to be on duty at that hour. For some time, however, the day nurses have been 10 minutes late, or even more, in taking up duty, and the visiting committee, having vainly adopted various methods with the object of inducing them to be punctual, decided to impose a fine of 6d. per minute on those who were late. This was so much resented by 26 of the day nurses, that they sent a letter to every member of the visiting committee, to the effect that unless the notice issued early in January, announcing a fine of 6d. per minute for being late in coming on duty in the morning were withdrawn within seven days, and a whole Sunday off duty were conceded once in three weeks instead of from 9 A.M. only, they should refuse to come on duty on February 11th, and should resign in a body. The asylum committee of the Sunderland Town Council having conferred with the medical superintendent, it was decided to withdraw the notice objected to, the nurses being willing that the demand for a full Sunday off once in three weeks should be separately considered. The affair seems rather trifling, but there were important principles at stake. There is no excuse for the unpunctuality of nurses, especially as at Sunderland their failure to be on duty resulted in the patients being left unattended for some minutes; and a threat to strike cannot under any circumstances be justified. We must add, however, that if a fine was supposed to be the only remedy, the amount proposed was excessive, and that the success of the nurses' ultimatum will hardly conduce to the maintenance of discipline in the asylum. It will be concluded that the committee having once yielded to an ultimatum will on another occasion be equally amenable to the same tactics.

### THE NURSES' DANCE AT LIVERPOOL.

In connection with the annual dance of the Liverpool City Hospital nurses, which takes place on Tuesday, it has been stated that a Matron's Committee decided that only professional men should be invited, and that therefore the male friends of some of the nurses are shut out. The Matron's Committee, it was added, justified their action on the ground that on a former occasion two men invited by the nurses attended in morning dress. As a matter of fact, however, there is no Matron's Committee, and the arrangements for the dance are left, as they have always been, entirely in the hands of the Deputy Chairman of the Port Sanitary Hospital Court. With regard to the institution, we are also authoritatively informed that instead of all the men invited belonging to the professional classes, as many, or more, are non-professional men, and that friends of the nurses have received invitations. It is true that on one occasion two male guests attended in morning dress, but this has in no way affected the issue of the invitations to the forthcoming dance. In these circumstances, we cannot see that there is any cause whatever for complaint, and it is a matter for regret that mis-statements should have obtained publicity in the local papers. It can readily be understood that if the dance is to become in any way a bone of contention the question may be raised whether a grant which is made every year by the Court solely in order that the nurses may enjoy themselves, is worth continuing.

### POOR-LAW INFIRMARY NURSES IN LODGINGS.

One of the drawbacks under which the nurses of Plymouth Workhouse Infirmary labour is that a proportion of the staff cannot sleep upon the premises because the accommodation is inadequate. The guardians, who are anxious to get rid of the superintendent because they attribute to her changes which they think are too frequent, do not appear to realise the objections to this state of affairs. They have lately given instructions to the architect to prepare plans for the erection of a women's general ward, but no attempt is to be made to provide the nurses with the proper sleeping quarters. Mr. Preston Thomas, Local Government Board Inspector, has expressed his regret that it is proposed to patch up the building, or make some little addition quite failing to meet the case, and he says that a nurses' home is an absolute necessity. "Five years ago," continues the inspector, "the nurses were very justly complaining about the accommodation, and things are much the same still. Many of the nurses are still out in lodgings, which is so obviously against good administration, that I do not understand the guardians allowing it to go on so long." The plea of the guardians is that they desire to save the pockets of the ratepayers; but we scarcely think that the ratepayers of Plymouth wish for the continuance of an arrangement which, as a make-shift, may be excusable, but as a permanent arrangement is indefensible. In fact, it reflects on the whole town, and we hope that the inhabitants will recognise the necessity of not allowing their reputation to suffer.

### SUNDERLAND WORKMEN AND DISTRICT NURSING.

The proceedings at the annual meeting of the Sunderland District Nursing Association this month were of a singularly satisfactory and significant character. It was stated in the official report, which showed the substantial balance of £175 15s. 10d. in excess of income over expenditure, that for the first time the working-men had contributed in 1904 from their yards and workshops, the subscriptions amounting to the handsome sum of £288 17s. 1d. Most of the workmen readily allowed a penny per month to be taken off their wages, and it was intimated that no fewer than 27 works have commenced to systematically support the association. For the present year there are promises of further support. In these circumstances, the members very properly elected four representatives of the workmen on the council, and the industrial classes have therefore the pleasure of knowing that those who are specially able to give effect to their views have a considerable voice in the management of the association. We heartily congratulate all concerned, including the indefatigable superintendent, Miss Rogers, on the valuable impetus which has been given to the movement by obtaining the warm financial assistance and the personal co-operation of the workmen of the town. In this respect Sunderland has set an example to the rest of England.

### THE RESIGNATIONS AT LEWES.

It appears that, according to the views of the present nursing staff at Lewes Union Infirmary, the reason why there have been so many resignations lately is that there has been a misunderstanding as to the conditions of appointment. The Lewes guardians having advertised for charge nurses, it has been concluded by successful applicants that a measure of work would be done by subordinates. This is not the case, however, and in order to prevent any misapprehension in future, the guardians have determined to advertise for a nurse only, as the staff really only consists of nurses and the superintendent nurse. It is impossible to state too clearly in an advertisement the precise terms of an appointment.

### SHEFFIELD INFIRMARY AND THE TRAINING OF MIDWIVES.

Like the Guardians of Aston, those of Sheffield Union have determined to act independently with reference to the recognition of their infirmary as an institution for the training of midwives, instead of joining in the Bradford memorial to the Central Midwives Board. We think that the Sheffield Guardians have rightly arrived at the conclusion that there is not any reason to suppose that, "applications for the recognition of Poor-law infirmaries as training schools will not be granted in cases where it can be proved to the satisfaction of the Central Midwives Board that the arrangements are such as would afford a training complying with the requirements laid down by the board." In other words, each application for recognition by the board must stand on its merits. It will be seen from our report of the last meeting of the Central Midwives Board that in future all Poor-law institutions will be inspected before the Board recognises them.

### MORE NURSES FOR NEWPORT INFIRMARY.

At the last meeting of the Guardians of the Newport Union, Monmouthshire, the infirmary committee presented a report to the effect that the existing staff of nurses at the Infirmary is insufficient to properly perform the necessary duties. They recommended the appointment of two additional probationers, and this recommendation was agreed to after considerable discussion. An application for an increase of salary by the superintendent nurse was also before the Guardians, who decided to refer the matter to the Finance Committee. The augmentation of the number of probationers to the requisite strength and the liberal treatment of the superintendent nurse will, no doubt, tend to render it more easy for the Newport Guardians to obtain as many nurses as they need.

### EARL SPENCER ON DISTRICT NURSING.

Presiding at the annual meeting of the Northamptonshire District Nursing Association, Earl Spencer, who as chairman submitted the report—which shows that there are no fewer than 15 local Associations affiliated with the county organisation—said that the question had now arisen whether midwifery cases could be combined with ordinary nursing. If the county Association had unlimited funds, they might have in every village independent nurses, but as they had not sufficient funds, he was afraid that if they tried to draw a distinction between midwifery and ordinary nursing cases they would find themselves in great difficulty. He was, therefore, in favour of combination. The ideal which the members of the Association had before them was, Lord Spencer said, to arrange that there should be in every village in the county a trained nurse, and he sincerely hoped that they would receive more support and be able to do more in respect to finding nurses for the sick of the county. A vote of thanks to Lady Wantage, Mrs. Fitzwilliam, and other ladies for assistance rendered to trained nurses was adopted.

### THE PENSION FUND AND SOUTHAMPTON NURSES.

On Thursday evening last week, by permission of Miss Mocatta, an address on the aims and objects of the Royal National Pension Fund for Nurses was given by the secretary, Mr. Louis H. M. Dick, at Grosvenor House, Grosvenor Square, Southampton. Upwards of 50 nurses were present, and in addition to those from Miss Mocatta's institution, the following representatives of hospitals and nursing institutions in Southampton attended, with members of their staffs :—Miss A. M. Monk, matron of the Eye Hospital ; Mrs. Varian, matron Hampshire Nurses' Institute ; Miss Clayton, superintendent of the local branch of Queen Victoria's Jubilee Institute ; Miss Dowbiggin, matron of the Poor-law Infirmary at Shirley Warren ; and Miss Vernall, night superintendent at the South Hants Hospital. The address, which was listened to with the closest attention, will doubtless result in the enrolment of many fresh members from Southampton and the neighbourhood.

### EAST LONDON NURSING SOCIETY.

A very successful entertainment in aid of the funds of the East London Nursing Society was given at Grosvenor House on Monday afternoon. In the absence of the president, Princess Christian, her place was taken by Princess Victoria of Schleswig-Holstein. The room, lent by the Duke of Westminster for the occasion, was filled to overflowing, among those present being some of the nurses working in connection with the society. The programmes, decorated with scenes from the life of the district nurse drawn by Mr. Lance Thackeray, were sold by a bevy of "baby nurses," in blue cotton frocks, caps, and aprons. The financial result of the sale of tickets, amounting to some £300, is the more satisfactory in view of the fact that the society is in urgent need of an additional £350 per annum in order to avoid the withdrawal of nurses from some very needy parishes.

### IRISH NURSES AND THE WOMEN'S SUFFRAGE QUESTION.

The members of the Irish Nurses' Association have been discussing the question of the enfranchisement of women. Ladies specially interested in the movement delivered interesting addresses, which were listened to with approval, and a vote of thanks to the speakers was proposed by Miss Huxley, president of the association. This was seconded by Miss Kelly, lady superintendent of Steevens Hospital, Dublin, and supported by Mrs. Manning, lady superintendent of the Dental Hospital, Dublin. At their last meeting on Saturday the Irish Nurses' Association greatly enjoyed a lecture by Dr. Travers Smith, visiting physician to the Richmond, Whitworth, and Hardwicke Hospitals.

### SHOREDITCH NURSING ASSOCIATION.

A representative list of speakers has been secured for the annual meeting in Shoreditch Town Hall of the Shoreditch and Bethnal Green District Nursing Association, which will take place on Thursday the 23rd instant. The Mayor of Shoreditch will preside, Lady Frederick Cavendish will represent the ladies, Mr. Claude Hay, M.P., the House of Commons, Prebendary Villiers the clergy of the Church of England, and the Rev. W. Cuff, Nonconformist ministers. The Princess Christian is president of this Association, which is one of the most important of district nursing organisations working in the crowded parts of the metropolis.

### THE NEW MATRON OF LARNE HOSPITAL.

The choice of a new nurse-matron of Smiley Cottage Hospital, Larne, which is announced in our list of appointments to-day, is remarkable for the fact that there were no fewer than 97 applications in answer to the advertisement which appeared in the columns of The Hospital. An excellent selection appears to have been made in the person of Miss Maclean, who was trained in two of the best of Scottish schools and has since had valuable experience in Scotland, which she will doubtless turn to the best possible account in Ireland.

### QUEEN'S NURSES AT BIRKENHEAD.

The feature of the report of the Birkenhead District Nursing Society for 1904, which was adopted at the annual meeting last week, was that though the work had been much heavier than in the preceding twelve months, there was no increase in the staff. The number of cases dealt with was 1,008 as against 901, and the number of visits paid was 27,302. Moreover, there were no fewer than 152 cases of pneumonia, or an average of three a week. The nurses, however, willingly responded to the extra pressure. This is very creditable to them, but if there is likely to be more work still this year, the question of augmenting the staff will need to be seriously considered. In these circumstances the slight reduction of the credit balance does not seem encouraging, but on the other hand the subscriptions were nearly £70 in excess of the previous year. Nothing succeeds like success, and the means of providing an additional nurse should be sought in a further advance in the source of income which shows such a gratifying upward tendency.

### THE MIDWIFE'S REGISTRATION FEE.

The Chard Guardians have wisely decided to pay the registration fee of one midwife in each parish who is willing to be registered. They have ascertained that there are midwives willing to be registered, but who plead that they cannot afford the fee. It may be observed in reference to this plea that after March 31 a midwife who is not registered cannot continue her work, and it is therefore penny wise and pound foolish not to fulfil its conditions. But guardians and other authorities may find it worth while to assist competent midwives by assuming responsibility for the payment of the fee.

### SHORT ITEMS.

The appointment of the new matron of the Royal Free Hospital will be made next week.—We are informed that Staff Nurses B. Rennie and L. F. A. Waller have been confirmed in their appointments in Queen Alexandra's Imperial Nursing Service, and that Miss Annie Buchanan Cameron has been appointed a staff nurse provisionally.—Among the congregation at St. Philip's, Stepney, on Sunday morning, when the Archbishop of Canterbury preached, were about 250 sisters and nurses of the London Hospital.—An enjoyable entertainment was given to the in-patients of the Cancer Hospital, Brompton, by Miss Cara Bennett and friends last week.

### The Nursing Outlook.

"From magnanimity, all fear above;
From nobler recompense, above applause,
Which owes to man's short outlook all its charm."

### UNITED WE TRIUMPH.

We have been carefully studying the whole of the Bills and proposed nurse-registration Acts of the American State Legislatures, together with the circumstances which affect nursing and public opinion in the United States. Later on we propose to deal with them in some detail because they present points which confirm to a great extent the position we have always taken up in regard to nurse registration. One cause why legislation has been so easily obtained in the United States is the comparatively recent date of State legislation in regard to the medical profession. The earlier Acts merely set up a register for nurses; in the later Acts the title of registered nurse is conferred and powers are taken to institute examinations and maintain a register, the costs of which are usually to be defrayed from the registration fees. No attempt has been made in America to interfere with any nurse wherever she was trained, or however little or much knowledge she might possess, who earned her living by nursing in whole or in part. The Acts merely confer upon the nurses who are registered the title of registered nurse and make it an offence for any other woman engaged in nursing to use this title. So long as a nurse, whoever she may be, refrains from representing herself as a registered nurse she can pursue the calling of a nurse and accept payment for her services from all who choose to employ her. We believe that this point is not sufficiently understood in this country, as it simplifies nurse registration and indicates the rock upon which the advocates of nurse registration in England have foundered more than once.

In the United States it is but just to point out that the nurses have, as a class, received a higher education than those in England. No one who is familiar with the quarterly and other journals issued by the nurses of the nurse-training schools in America can doubt this fact. Then too the "American Journal of Nursing" is undoubtedly from a literary point of view probably the best edited publication of the kind in the world. It has not as yet so organised its news columns as to be able to give the true position of nursing matters in this country; but, so far as American nursing is concerned and the many general questions in which the nursing profession is interested, its columns reflect the greatest credit upon those who are responsible for its publication. This advantage of higher education has made American nurses realise, that once the title of registered nurse is established by law, they will be able, if they are united, to gradually educate the public to understand how and where to obtain the services of a really efficient woman to nurse them in the hour of sickness. Conscious that their own desire to secure that every registered nurse shall be highly qualified and self-respecting is based upon principle, they rightly believe that, by uniting their forces, everything else that is desirable must come to them in due course. American nurses too have had the great advantage of being supported, in their aspirations to secure the best, by the authorities of the more important nurse-training schools. Unfortunately, so far in England, several nurse-training schools have declined to co-operate and have discouraged, if they have not even prevented, their nurses from co-operating together too. All this is bad for the public, for the nurses, and for the schools.

It must necessarily take time to bring about complete co-operation between the nurses who can most worthily represent their profession in England, but never before have the inducements to co-operate been so strong as they are to-day. The best of the nurses are fully conscious of the importance of being united, and as the nurse-training schools have declined to co-operate with the nurses in securing this result, it is essential that united action should be taken through a neutral and independent body in which every nurse can have justifiable confidence. That is why the establishment of a Central Council of Nursing Education through the independent and strong initiative of great merchant princes like Lord Rothschild, Lord Revelstoke, Mr. E. A. Hambro, and several governors of the Bank of England, should receive the spontaneous and united support of every intelligent nurse in England to-day. It is not possible for us to give the plan on which the Central Council of Nursing Education will be established, because that has not been finally determined. The important point, however, is, that Lord Rothschild and the other merchant princes offer all English nurses, and indeed all nurses in the British Empire, a neutral platform upon which they can unite, where they can secure full representation and a guarantee, that every interest they may have will be safeguarded and secured, as a vital principle. The members of the Council and of the Examination and Consultative Boards must in fact be representative of every interest in the nursing world. Neutral but representative as this Council and these Boards must be, the sneer of lay control cannot be launched against Lord Rothschild's organisation. Thus from the outset it must be freed entirely from all personal interests, whether institutional or individual, for personalities have been the curse of nursing affairs in England in the past. In such circumstances every educated and intelligent nurse, who has a love for her profession, will scarcely need our counsel to decide, at once, to unite with her sisters in support of Lord Rothschild and his society. By uniting on the Rothschild platform English nurses may once and for all be guaranteed the independence and security which they have felt the need of so often in the past. Nurses already owe much to the Rothschilds, but their obligations are likely to grow apace, when the Society for Promoting the Higher Education and Training of Nurses has settled down to the business of organisation, and the provision of complete security for all competent nurses.

### Lectures Upon the Nursing of Mental Diseases.

By Robert Jones, M.D.Lond., B.S., F.R.C.S.Eng., M.R.C.P.Lond., Resident Physician and Superintendent of the London County Asylum, Claybury.

LECTURE XXIV. (*concluded from page 259.*)

The instruments employed for enemata are of various kinds: (*a*) *The glycerine Syringe* is made of glass or vulcanite, and it holds any quantity from one teaspoonful (which is the usual dose) to an ounce. (*b*) *The "Higginson syringe,"* which is used for relieving the bowels, and, by attaching a special tube, for vaginal injections. It is simple in use, the bulb being squeezed and released alternately. The fluid flows only in one direction, and as the instrument is usually made of indiarubber the elasticity of the material permits a continuous and even flow. (*c*) *A bulb* or *india-rubber bottle*, capable of containing from four to six ounces, called the "ball syringe," with a short ivory tube screwed on the end of the bottle, for administering nutrient enemata, which, as stated, do not exceed four ounces in quantity. Two other kinds of syringes which, although not used for enemata, are in frequent use and may be referred to here—viz. (1) *the hypodermic syringe*, a small glass tube, with register for measuring drops marked upon it. A fine hollow needle is at one end, and the handle of the piston is at the other. When charged, the contents are injected under the skin; and (2) *the ordinary brass syringe*, with an ivory or brass nozzle, used for washing the ear passage and for cleaning wounds, or in removing dressings.

*Baths.*—(*a*) *Hip-baths*, for diseases within the pelvis. These baths should not be too full, and during the sitting a blanket should be thrown over the body. (*b*) *Ordinary hot baths*, ranging from 100° upwards to 110° F. Hot baths may be used with ice to the head. (*c*) *Tepid baths*, from 80° to 90°. (*d*) *A warm bath* should be about the temperature of the body; and (*e*) *a cold bath* the temperature of the air—colder, of course, in winter. (*f*) *Continuous baths* for hours, or days, or even weeks, at a degree or so above the body-temperature, used for acute mania and for surgical cases, such as burns, etc. (*g*) *Graduated baths*, for high fever, *i.e.* gradually reduced whilst the patient is in the bath from 100° to 70° or 60°, or until the temperature of the patient is lowered to about normal. These have been used with success in the *status epilepticus*; or ice may be applied in an ice-cap and may be used without the bath. (*h*) *Hot-air baths*, given in kidney disease to stimulate the action of the skin; also in cases of melancholia in young persons, to improve the mental condition. (*i*) *Turkish baths* have this effect. (*j*) *Russian* or *vapour baths*, when steam is used instead of air. (*k*) *Mercurial baths*, when a patient well covered with a blanket sits on a cane-bottomed chair, under which a dish of calomel is evaporated over a spirit-lamp, the vapour of the steam being used at the same time, and the bath lasting for about 20 minutes.

The cold and hot wet-pack have already been described. These and the half pack and tepid sponging are used in cases of restless excitement with or without fever, and the sponging especially for cases of high fever, when the skin is dry. Douches, nasal or vaginal, are used as directed.

*Cases of confinement* occur not infrequently among patients in asylums for the insane. In a little over two years at Claybury Asylum 30 women, whose insanity took place during pregnancy, gave birth to infants at varying intervals after admission. There is generally but little warning by pains of an approaching confinement; the pains are short, and labour may occur precipitately, the child being born within a short time of the initial pain. The fullest preparations for confinement should therefore be made for any case of insanity complicated with pregnancy as soon as the patient is received, and absolute cleanliness is imperative in every respect. There must on no account be any patient near a child-bed case that has suffered from any discharges, and the nurse should not have been near any case of fever or of any infectious cases. When the pains begin, the bed should be prepared, a new mackintosh sheet (covered with a fresh draw-sheet) being arranged upon it, and all clean bed-clothes furnished for the case. Three stages of labour are described: the first of dilatation of the parts for the passage of the child; the second stage being the birth of the infant; and thirdly, the expulsion of the after-birth or placenta, after which the binder is fixed. There are certain fixed rules for the nurse to observe which emphasise the necessity for cleanliness in every detail. All metal or glass instruments are to be boiled; all rubber or gum-elastic ones kept disinfected by being placed in antiseptic solutions, and the greatest care is to be taken with the hands, fingers, and nails. All soiled articles must be immediately removed, and soiled pads burnt after use. During the first few days the vagina is to be douched night and morning with *antiseptic* solutions as may be directed, and the patient kept perfectly quiet. Antiseptics are generally chemicals which arrest or prevent putrefaction, without destroying the germs, whereas disinfectants destroy the germs themselves as well as arrest putrefaction. The antiseptics used for douches are usually carbolic lotions 1 to 60, permanganate of potash or Condy's fluid, corrosive sublimate 1 to 5000, a saturated solution of boracic acid, tincture of iodine 1 teaspoonful to a pint of water, a weak solution of chloride of zinc, and such preparations as izal and other coal tar products. The temperature of the patient is taken and recorded twice daily, and reports made as to any discharges, and also as to the state of the breasts, the bladder, and the bowels. In the insane the baby is not permitted to be nursed by the mother, and special directions are given for its care and feeding. Attention to the state of the perineum in first cases, and also to the condition of the breasts is very necessary. With careful and absolute cleanliness, and close attention to details, every case should do well; but many of the insane are very difficult to keep chemically or bacteriologically clean. Attention to the infant includes care in regard to the navel, the eyes, the groins, micturition, the state of the bowels, and the appearance of any rash.

*Poultices*, which are means of applying moist heat locally, should always be made as hot as can be borne. If too hot, or if used in the case of a "lost" patient, flannel must be placed next to the skin. Poultices are of various kinds, charcoal being used for ulcers and bed-sores, or offensive discharges, and yeast for foul wounds. A mustard plaster made with mustard and cold water is not always a safe remedy for weak-minded persons with a feeble circulation, as the redness of the skin caused by the mustard may result in a sloughing sore. Linseed or bread may be used for making poultices. When used to cover the whole chest, back and front, as in cases of pneumonia, they are called "jacket poultices." Fomentations and stupes are used as hot as can be tolerated. Fomentations are made by placing an open towel over a basin. A piece of flannel a yard square, and closely folded, is then placed upon the towel in the basin and boiling water poured upon them from a kettle. The towel is folded over the flannel and each end squeezed so that the flannel and towel are wrung free from water; both are then carried to the patient. The towel is now unrolled and the flannel is ready for use as a fomentation. In using belladonna paint the glycerine extract over the part first.

and then apply the fomentation, but in a poppy fomentation the poppy-heads are crushed and boiled, the fomentation being made of the water thus used. Turpentine is at times sprinkled over a hot fomentation, but will soon blister if the fomentation is kept on too long. Turpentine fomentations are used for abdominal pain. Spongio-piline may be employed in the place of flannel if desired. Counter-irritants, such as mustard, blistering fluid, and liniments, are also used for relieving pain. When blisters are thus formed they should be pricked, but the fluid let out must not be allowed to run over the skin as it may cause further blistering. Sweet oil or simple boracic ointment can be used as a subsequent dressing. Leeches and cupping are now rarely used. Ointments are often prescribed as applications to raw surfaces; they are medicated lard or oily preparations. Inhalations are not easy of application for mental cases, but where their use is possible they are efficacious for bronchial affections, and they afford much relief for colds such as nasal and other catarrhs. The bronchitis kettle is a familiar means of applying moisture in the shape of vapour or steam to inflamed breathing tubes.

*Death.*—Before concluding these lectures, perhaps a word or two may be fittingly offered upon the final duties of the nurse when death occurs—a sad and regrettable consummation to the ministrations of the nurse and to the feelings of the friends. When the patient dies the eyes should be closed by gentle pressure with the fingers for a few minutes, or a small weight, such as a penny, may be placed upon the closed eyelids. The limbs should be straightened out carefully, and a bandage applied under the lower jaw to support it. The arms are placed at the side and the hands across the chest; the lower limbs are kept in position by a bandage connecting the great toes. The clothing should be removed, if possible, soon after death and before *rigor mortis* sets in, and the body is thoroughly washed, after which a clean bedgown is placed over the remains.

### The Arrangement of Constant Hot Irrigation to an Injured Leg.

### EXAMINATION QUESTIONS FOR NURSES.

THE question was as follows:—Describe how you would arrange constant hot irrigation to an injured leg.

### FIRST PRIZE.

After arranging the patient's bed-clothes so that the other parts of the body were well covered and the bed neat, I should expose the leg to be irrigated. After raising it upon a pad or pillow to form a slope, I should place a mackintosh underneath the leg, but over the pillow, arranging it so that it hung over the side of the bed, and turning the sides over scoope fashion, so that the fluid would escape into a receptacle, such as a pail or foot-bath, placed beneath.

After removing the dressings and placing an antiseptic pad over the wound, if there be one, for the time being, I should place a cradle over the leg, from which I should suspend anything capable of holding about one pint of fluid or a little more, such as a child's pail, tin, or enamelled can with a handle over the top if possible, making one with stout string if there were none. This should be filled with the lotion ordered, much hotter than required to allow of its cooling. After cutting some strips of lint or flannel, I should place one end of each into the lotion, allowing the other ends to hang over the side immediately over the surface to be irrigated, causing a constant dripping upon it. If there be a wound the pad should now be removed, leaving it exposed.

I should have a jug of lotion mixed ready and much hotter than required, with which I should keep refilling the receptacle as it emptied from time to time, keeping it full and also maintaining the required temperature. The bath beneath I should empty occasionally as it required.

If the wound was not in the foot I should put on a woollen sock, and if the patient felt cold I should place a hot water bottle against the foot.

Stout brown paper, or even several sheets of newspaper, could be used instead of the mackintosh to carry away the fluid. A foot-stool or wooden box, placed by the side of the leg on the bed with a deep overhanging dish placed upon it, could be arranged in the same way and answer the same purpose as the cradle, etc., provided it was sufficiently higher than the leg. "CENTRAL."

### SECOND PRIZE.

Arrange the patient so that the injured limb is on the outside edge of the bed, and place a piece of mackintosh or American cloth beneath it, letting one end hang over the side of the bed as much as possible. Put a hot-water bottle to the exposed foot, and cover with a sock. Have ready some boiled white rag or lint, plenty of hot and cold sterilised water, and a piece of boiled lamp wick. Get a table—an ordinary round one with a centre stem I find answers the purpose best—and push this well over the bed. Fill a clean jug with the water, making it the required temperature by the aid of a bath thermometer, and allowing a degree for its cooling in transit along the wick. Now put the lint or rag over the injured part, place one end of the lamp wick into the jug, letting the other end hang directly over the injury, and in this way a constant hot stream of water is obtained. The temperature can easily be kept to the doctor's orders by adding hot water as it cools, and if a covered jug is used the heat will be retained longer. If the leg be raised by placing two pillows under it, one above and one below the injury, and the mackintosh arranged in a groove, there will be no danger of the bed getting wet. It is, of course, necessary to place a bath under the bed to catch the water from the mackintosh.

"GARDE-MALADES."

### THE PRIZE WINNERS.

"Central" is again successful in gaining the first prize, and "Garde-Malades" takes the second. The papers this month are satisfactory and show a marked improvement to the answers sent in thirteen months ago to the same question. The reason "Central" and "Garde-Malades" are awarded prizes is that they have directed their attention to explaining what they would do in cases where appliances are few and circumstances—lack of means or the long distance from a town perhaps—would entirely prevent resorting to the many excellent arrangements primarily concerned with tubing, mentioned by several of the candidates. These answers are good, many of them showing considerable thought and ingenuity, but the examiner wished you all to imagine yourselves kind of Robinson Crusoes on a desert island where tubing-clips, nozzles, and funnels are not!

### HONOURABLE MENTION.

This is awarded to "Thatch-house," "Aubrey," "Lanoitan," and "Brum."

### AN ERROR TO BE AVOIDED.

Never make the desired fall to carry off the lotion from the head to the foot of the bed. It is always difficult to keep a patient from slipping down at the best of times, without giving him an unnecessary push further down by this arrangement. Moreover, it would give a dragging sensation to the injured limb. On all occasions let the waste lotion run off over the side of the bed.

### QUESTION FOR FEBRUARY.

If called upon to nurse a case of diphtheria in a house where means are very limited, and appliances such as are used in hospitals not to be obtained, what arrangements should you make?

Rules.

The competition is open to all. Answers must not exceed 500 words, and must be written on one side of the paper only, without divisions, head lines, or marginal notes. The pseudonym, as well as the proper name and address, must be written on the *same* paper, and *not on a separate sheet.* Papers may be sent in for fifteen days only from the day of the publication of the question. All illustrations strictly prohibited. Failure to comply with these rules will disqualify the candidate for competition. Prizes will be awarded for the best two answers. Papers to be sent to "The Editor," with "Examination" written on the left-hand corner of the envelope.

In addition to two prizes honourable mention cards will be awarded to those who have sent in exceptionally good papers.

N.B.—The decision of the Examiner is final, and no correspondence on the subject can be entertained.

Any competitor having gained three prizes within the current year shall be disqualified from taking another until 12 months shall have expired since the first prize was gained.

### Central Midwives Board.

An adjourned meeting of the Central Midwives Board was held on Thursday, February 9th, to consider and deal with a report of the Standing Committee upon a number of applications for approval from institutions, as training schools, medical practitioners, as teachers under rule C.I. (3), and midwives under rule C.I. (2).

There were present: — Dr. Champneys, chairman, Dr. Cullingworth, Mrs. Latter, Miss Paget, Sir William Sinclair, and Mr. Parker Young. Agreement with the resolutions of the Standing Committee being moved from the chair, this was carried, with two dissentients, Sir William Sinclair and Mr. Parker Young, the former protesting that the Board had been guided by no principle whatever for months past in the matter of approving applications for recognition, and had recognised such unsuitable persons and places that he could see no reason why any should be refused. Dr. Cullingworth pointed out that if any unsuitable applications had been accepted it had been from no want of care on the part of the Board.

The report dealt with a number of institutions, which (with the exception of the Hospital for Soldiers' Wives and Children, Devonport, approved, subject to conditions), were postponed for inspection or inquiry.

The following medical practitioners were approved as teachers under Rule C.I. (3): Gerald Cree, Major, R.A.M.C., M.R.C.S.(Eng.), L.S.A. (Lond.); John S. Fairbairn, M.B., B.Ch. (Oxon.), F.R.C.S. (Eng.), M.R.C.S. (Lond.); E. Falkier Hill, M.B., Ch.B. (Vict.), M.R.C.S., L.R.C.P.

The following certified midwives were approved under Rule C.I. (2). — Matilda Jane Atthill, Lily Blackburn, Ellen E. Boast, Beatrice A. Ellis, Amy Evans, Anna Hill, Eliza Ann Hodginson, Margaret McL. Knipe, Elizabeth Nicholls, Edith Olliff, Margaret E. Partridge, Maude B. Welch.

Special Meeting.

A special meeting of the Board was held immediately after the adjourned meeting, to consider the principles which should be followed in recognising Poor-law infirmaries as training schools for midwives. Miss Wilson, who was unable through illness to attend, wrote expressing her strong opinion that until the Board had before them a certain circular of the Local Government Board dealing with midwifery in these institutions, for which she had made application without success, it would be impossible to proceed further in this matter. Miss Paget also thought this most important; apparently practice was sometimes directly opposed to the standing orders, and she considered that it was essential to know the conditions under which midwifery was carried on in Poor-law institutions before deciding whether the training could be trusted.

A good deal of discussion followed upon a resolution of Dr. Cullingworth to the effect that all Poor-law institutions should be inspected before approval. Finally it was carried unanimously:—"That all Poor-law institutions, before recognition by the Board, be made the subject of investigation and report. A rider, moved by Sir William Sinclair and seconded by Mr. Parker Young, "that a report be asked for from the chief medical officer in each case, and the local supervising authority invited to inspect and report upon such institutions," was carried after considerable discussion.

Mrs. Latter voted against this, on the ground that an independent report would be infinitely more valuable, and Miss Paget pointed out how utterly unsuitable in many cases the local supervising authority would be to make any such inspection. The meeting was adjourned to February 16th.

### Association for Promoting the Training and Supply of Midwives.

The annual meeting of the Association for Promoting the Training and Supply of Midwives was held at the Caxton Hall on Tuesday morning.

Mr. H. Cosmo Bonsor presided, and among those present were Lady Brassey, Lady Prideaux, Mrs. Travers, Mrs. Percy Boulnois, Lady Priestley, Dr. Boxall, Miss Broadwood, Mrs. Norman Murison, Miss R. Paget, Mr. A. L. Leon, Mrs. Charles Schwann, and Mrs. Wallace Bruce (chairman of the Executive Committee). Among the new members of council announced were the Duchess of Marlborough, the Viscountess Knutsford, Sir Harry Poland, K.C., Lady Priestley, Sir Thomas Smith, M.R C.S.; Mr. E. A. Brotherton, M.P.; Mr. Wallace Bruce, L.C.C.; Mr. Arnold Gabriel, and the county medical officers for Northamptonshire, Staffordshire, Surrey, the West Riding of Yorkshire and Glamorganshire. Lord Brassey and Mr. Charles Schwann, M.P., have consented to become vice-presidents of the Association. Mrs. Wallace Bruce moved the adoption of the annual report, and dwelt on the important points of work undertaken during the year.

The report gives a review of the work of the year and of the meetings held. With regard to the training of midwives, the association has already trained eight, five of whom are from the country and three Londoners.

On this subject the report says:—

Our best candidates have come through the recommendation of friends, and we greatly hope to hear of many more in this way. Vacancies are engaged for 1905 at the East End Mothers' Home, the York Road Hospital, and the Clapham Maternity Hospital, in addition to Skeffington Road, and will be constantly added to as candidates present themselves and funds increase. Fees for examination and partial travelling expenses have also been paid in the cases of two practising midwives from the West of England who were desirous of going in for the L.O.S. exam., but could not meet the expense. Without this help they would have had to enrol themselves simply as *bona fide* midwives; by means of our help they have been enabled to become properly qualified and take a good position.

Cases frequently come to our notice in which the 10s. fee for enrolment is prohibitive; to a poor woman in practice at very low fees, where good reference is given as to character and real poverty, the association is ready to pay a part or the whole of the fee, to enable her to take advantage of

Clause 2, and come upon the register before April next, and continue to be called a midwife.

The association will have several midwives ready for work after the next L.O.S. examination, and the secretary is prepared to recommend these midwives for district work among the poor, if application is made to her.

### Re-organisation of the Training School at the General Lying-in Hospital.

In order to meet to some extent the increasing demand for trained midwives and monthly nurses, consequent on the operation of the Midwives Act, four classes of pupils are now received for training at the General Lying-in Hospital, York Road, Lambeth. These are: (1) Hospital-trained midwives (for those who have had sufficient training and experience in general or monthly nursing); (2) District-trained midwives (for those who have had sufficient previous training and experience in nursing); (3) Hospital-trained monthly nurses; and (4) District-trained monthly nurses (for those who have had sufficient previous training and experience in general nursing). In all cases the period of training may be extended by arrangement. The course of training for midwives, whether in the wards or in the district, is in accordance with the rules framed by the Central Midwives' Board, and with the regulations of the London Obstetrical Society for their certificate.

Pupil-midwives training mainly in the hospital must either have received at least eight weeks' training in the hospital, and have obtained a certificate as a hospital-trained monthly nurse; or eight weeks' training as a pupil district nurse, with certificate as a district-trained monthly nurse; or must produce satisfactory evidence of sufficient training and experience in general or monthly nursing elsewhere. The course of instruction and training comprises attendance at lectures by the physicians; classes by the house-physician, the teacher of midwifery and the resident midwife, in the hospital; attending labour cases in the delivery-rooms under the supervision of the resident midwife and the house-physician, and at the patient's own home under the supervision of the district midwife attached to the out-patient department in the immediate vicinity of the hospital. During attendance on cases outside the hospital, the pupil midwives sleep and take their meals in the training home for district midwives and nurses.

Pupil midwives, before entering for training in the out-patient department, must either have received at least eight weeks' training as a pupil nurse in the hospital, or as a pupil district nurse, and have obtained a certificate as a hospital-trained monthly nurse; or a district-trained monthly nurse; or produce satisfactory evidence of sufficient training and experience in general or monthly nursing elsewhere. Failing which, pupil district midwives are required to undergo instruction and training in the hospital for four weeks (counting as part of the obligatory three months) as probationer nurses. The course of instruction and training comprises attendance at lectures by the physicians; classes by the house-physician and the teacher of midwifery, in the hospital; and classes conducted at her own house by the district midwife to whom the pupil is attached; the conduct of labours in the patients' own homes under the supervision of the district midwife; or, in the case of probationer nurses, assisting at such cases in the delivery-rooms in the hospital; and the nursing of lying-in patients in their own homes under the supervision of the district midwife; or, in the case of probationer nurses, in the lying-in wards, under the superintendence of the matron.

For the purpose of a residential training home, a house immediately opposite the hospital has been fitted up, through the kindness of Dr. Robert Boxall, Mrs. Boxall, and their friends, to accommodate six nurses and two midwives. Two large bedrooms have been curtained off into cubicles, three in each for the nurses, and a double-bedded room is set apart for the midwives. There is, in addition, a cosy dining and sitting-room, and a bath-room, cloak-room, etc., have been fitted up in the basement. Comfortable and well-furnished apartments are provided for the Home Superintendent, Miss Francis, and the Resident District Midwife, Miss Rae. The walls are colour-washed in light buff; the furniture is conveniently compact; cork carpet has been laid on the floors, and electric light is installed throughout. Since July, when the scheme came into operation, 66 midwives and one nurse have been trained in the district, in addition to 33 midwives and 94 nurses who received instruction in the wards of the hospital. Dr. Boxall has further shown his interest in the training school by the establishment of a valuable midwifery scholarship.

### Everybody's Opinion.

[Correspondence on all subjects is invited, but we cannot in any way be responsible for the opinions expressed by our correspondents. No communication can be entertained if the name and address of the correspondent are not given as a guarantee of good faith, but not necessarily for publication. All correspondents should write on one side of the paper only.]

### THE FEES OF MIDWIVES.

"Certified Midwife" writes:—For the benefit of many like myself, would it not have been possible for the Central Midwives Board whilst preparing and getting the new Act passed, to have added the regular fee to be charged by midwives generally, say perhaps 10s. 6d., the fee which for the last 12 years I have charged for delivery and attendance upon mother and child for about nine days? In the course of my practice I have found many women object to this fee, saying that the Gamp type charge was 7s. 6d. or even 5s. 6d. This does not pay a midwife for her arduous duties, often including tedious labours. I should like to hear what other midwives have to say about it, as I think it is a subject likely to crop up more than ever now. Also I notice that the new register has no column for the "Temperature," which I always thought was very necessary, neither the "Syringe," which could, of course, be given or omitted as the midwife saw the patient required.

### WOMEN INSPECTORS OF MIDWIVES.

L. M. B. writes:—A letter written by a recent correspondent regarding the employment of inspectors who only hold qualifications in midwifery should be taken up by all who are fully-trained certificated nurses. I cannot resist writing a few words concerning the injustice of employing other than nurses with a three years' general training and an additional maternity certificate. We, as nurses, spend three years or more in a general hospital, at no small cost. We study during that period to become acquainted with all kinds of ailments and the numerous ways and means to help as nurses to alleviate these and then leave the hospitals for district or private nursing to meet with those who, after a much shorter period—even as little as three months—possess only a meagre knowledge of the rudiments of maternity nursing. What is worse still these women are taking the responsible posts as district nurses and also receiving the same salaries. The latter is not hearsay but actual knowledge. I am a fully-trained nurse of several years' experience in district nursing, and in many neighbouring districts I have met the nurse of the district, who is only maternity trained. I think that an appeal should be made to all committees and persons responsible for engaging district nurses to see to it that only those who are fully-trained nurses should be elected to the posts.

### A MISCHANCE WITH THE CATHETER.

"Inquirer" writes: I should like to make known through your columns an unfortunate, and, I hope, unusual experience I have lately met with, with a view of ascertaining whether others of your readers may have heard of or had a similar mischance. In passing the catheter (a glass one) a week ago on my patient, an elderly lady, when withdrawing the instrument I noticed that the eye end was broken off to about the length of an inch. Fortunately the break was straight across, and not jagged, and the end of the removed portion blunt. There was no pain felt, the patient in fact, being unaware of what had occurred. The doctor, after making a slight but unsuccessful effort to remove it, decided to leave it in the bladder so long as no untoward symptoms appeared, and so far, I am thankful to say, there has been no indication whatever of anything abnormal. I have heard definitely of one other similar case, and vaguely of one or two others, and should be interested to hear whether such a thing has happened comparatively often, and with what result. I had used the same catheter for six months, and was most careful in dealing with it not to knock it, and always left it to cool after boiling, and therefore cannot account for the accident. The patient was perfectly still during the procedure. Feeling there was the possibility of such a thing happening, and knowing of the other case, made me extra careful.

### Appointments.

[No charge is made for announcements under this head, and we are always glad to receive, and publish, appointments. The information, to insure accuracy, should be sent from the nurses themselves, and we cannot undertake to correct official announcements which may happen to be inaccurate. It is essential that in all cases the school of training should be given.]

Alexander Hospital, Queen Square, Bloomsbury.—Miss Muriel Ray has been appointed assistant matron. She was trained at St. Mark's Hospital, City Road, London, and the Great Northern Central, and has since been sister at St. Mark's Hospital.

Bolton Infirmary and Dispensary.—Miss Isabella Milne has been appointed sister. She was trained at the Royal Hospital for Sick Children and at Leeds General Infirmary.

British Home for Incurables, Streatham.—Miss Bertha E. Hutchison has been appointed sister. She was trained at University College Hospital, and was for two years in South Africa as a member of the Army Nursing Service Reserve. She subsequently became night superintendent at Southwark Union Infirmary.

City Isolation Hospital, Nottingham.—Miss May Stamthorpe has been appointed night sister. She was trained at Middlesbrough Sanatorium and Huddersfield General Infirmary, and has since been sister at Stanley Hospital, and sister at Mill Road Infirmary, Liverpool.

Dean House Workhouse, near Holmfirth, Yorkshire.—Miss Harriet Ann Sidebottom has been appointed charge nurse. She was trained at the Leicester Borough Asylum, and has since been nurse at the West Riding Asylum, Wakefield.

Hospital for Incurable Children, Hampstead.—Miss Agnes Davies has been appointed staff nurse. She was trained at Fulham Infirmary, and has since been staff nurse at Shoreditch Infirmary, and staff nurse at St. Luke's House for Incurables, Bayswater, London.

Jessop Hospital for Women, Sheffield.—Miss Frances J. May has been appointed staff nurse. She was trained at the Union Infirmary, Kidderminster, and the East End Mothers' Home, London. She has since been assistant nurse at the Epsom Cottage Hospital and assistant nurse at Bideford Dispensary and Infirmary.

Liverpool Stanley Hospital.—Miss Mary Dight has been appointed sister. She was trained at the Sheffield Royal Hospital, and was afterwards attached to the Victoria Nurses' Institute, Bournemouth.

Mercers' Hospital, Dublin.—Miss Amy Butler has been appointed matron and lady superintendent. She was trained at the Rotunda Hospital and Sir Patrick Dun's Hospital, Dublin. She has since been housekeeper and sister-in-charge, of the gynæcological department at the Rotunda Hospital, Dublin, and sister-in-charge of the medical wards at Sir Patrick Dun's Hospital.

Scottish National Institution, Larbert, Stirlingshire.—Miss Helena M. S. Thornton has been appointed nurse in charge of the new sanatorium. She was trained at the Brownlow Hill Infirmary, Liverpool, and the City Hospital North, Liverpool. She has since been nurse at the Town's Hospital, Glasgow, and at the Royal Infirmary, Edinburgh, and has been engaged in private nursing.

Smiley Cottage Hospital, Larne.—Miss Jessie Maclean has been appointed nurse matron. She was trained at the Royal Hospital for Sick Children, Edinburgh, and at the Aberdeen Royal Infirmary, where she was afterwards staff nurse. She has also been charge nurse at Falkirk Infirmary, taking matron's duty when necessary.

Stockport Infirmary.—Miss Alice Mary de Ville has been appointed matron. She was trained at the Victoria Hospital, Burnley. She has since been night sister at Princess Alice Hospital, Eastbourne, night superintendent at the Royal Infirmary, Halifax, sister at the Royal Hospital, Portsmouth, and assistant matron at the Royal Infirmary, Bradford.

### Presentations.

St. John's and Greenham District Nursing Fund.—A silver tea service and a cheque for £28 has been presented to Miss Caroline Freeman, upon her resignation of the post of district nurse for St. John's, Newbury, and Greenham parishes, which she held for 11½ years. This is the second presentation which has been made to Miss Freeman, the first having been a bicycle and purse in 1901.

### Death in Our Ranks.

We regret to hear of the death, on February 8th, of sister Agnes Barns, of St. Mary, Islington, Infirmary, after a short illness. Miss Barns was trained at this infirmary, was promoted to be staff nurse in 1904, and on January 4th, 1905, was again promoted to be ward sister. Many wreaths were sent at the funeral in token of the esteem and deep regret of the staff.

### To Nurses.

We invite contributions from any of our readers, and shall be glad to pay for "Notes on News from the Nursing World," or for articles describing nursing experiences at home or abroad dealing with any nursing question from an original point of view according to length. The minimum payment is 5s. Contributions on topical subjects are specially welcome. Notices of appointments, letters, entertainments, presentations, and deaths are not paid for, but we are always glad to receive them. All rejected manuscripts are returned in due course, and all payments for manuscripts used are made as early as possible after the beginning of each quarter.

### Echoes from the Outside World.

### The King and Parliament.

On Tuesday the King, who was accompanied by the Queen, opened Parliament in person. The ceremony was one of great brilliance, and all along the route, both going to Westminster and returning, their Majesties were received with great enthusiasm by the spectators. The King read the gracious Speech from the Throne in a distinct and audible voice. It made reference to the visit of the King and Queen of Portugal, the war in the Far East, the condition of the Balkan Peninsula, the Convention with France, the Expedition to Thibet, and the Scottish Church dispute. Measures are promised dealing with alien immigration, the problem of the unemployed, the adulteration of butter, and other matters. On Saturday his Majesty received at Buckingham Palace Mr. Albert Hardwick and bestowed on him the Royal Albert Medal, first class, for an act of courage in rescuing an elderly lady, who on December 21st last, fell from a platform at Finsbury Park Station in front of an incoming train. The King also bestowed a second-class medal of the same order on chief stoker Alfred Stickley of the Royal Navy who, in January 1904, showed great bravery and sustained severe injuries in dealing with an accident to the machinery of the torpedo-destroyer *Success*. By the express desire of the King, two aged sisters who had been deprived of the privilege of selling milk in St. James's Park, have been granted a new site near the Horse Guards Parade where they will be able to sell milk as long as they live.

### The Queen and the People.

The Queen received at Buckingham Palace last week the Rev. W. Carlile, hon. chief secretary of the Church Army, and at her Majesty's desire he laid before her a statement of the operations of the organisation. When he told her Majesty that her gift of £50 at Christmas had stimulated others to provide 6,000 dinners for those who otherwise would have had none, she said: "I was deeply touched by the accounts I read of the affectionate sympathy with which the poor dear people received the mention of my name." Referring to the King's labour tents she said, "My heart goes forth to those poor fellows. I am so glad you try to help them." Her Majesty made special inquiries after the prisoners' wives and children, and also concerning the respectable married men with wives and families, who were being relieved by the Mansion House Fund. In conclusion, she sent affectionate greetings to all the earnest workers of the Church Army throughout the world, encouraging them to live and labour for the good of others.—The little invalid daughter of a Camberwell working man wrote a letter to the Princess Victoria last week in which she said "I went to church this morning to secure a blessing before going into Brompton Hospital. At church prayers were said for you, and I am sorry you are so ill, as Vicky is my name also. When I get into the hospital I will pray for you, and I hope you will think of me sometimes." Two days after a Royal messenger drove up to the house of the family, and presented to the child's mother a beautiful basket of flowers, stating that they were from the Queen, who had also sent a letter. The letter was as follows:—"Miss Knollys is desired by Princess Victoria to thank her little namesake very much for her nice letter, and to say how much her Royal Highness hopes and prays that God will soon make them both well again. The Queen sends a few flowers which she thinks little Victoria will like to have by her bedside."

### The New Lord Warden of the Cinque Ports.

Lord Curzon having resigned the office of Lord Warden of the Cinque Ports, the King has approved the appointment of the Prince of Wales. This means that the historic title will be maintained; but it is announced that Walmer Castle will no longer be used as a residence for the Lord Warden. The rooms as well as the ramparts and the gardens will, subject to regulations, be thrown open to the public on and after May 1st. Walmer Castle was built by Henry VIII., but the office of Lord Warden is of much greater antiquity. Among the Royal holders of it were Henry V., when he was Prince of Wales, Richard III., when he was Duke of Gloucester, Henry VIII. when he was Duke of York, and James II. when he was Duke of York. The office was also held by Prince George of Denmark, consort of Queen Anne.

### Russia and the War in the Far East.

There is little news of importance from the seat of the war. Vladivostock is declared to be in a state of siege, and a German steamer with warlike stores for the besieged has been captured by the Japanese. The number of Russian dead buried by the Japanese in the Hei-kau-tai region up to last Friday is said to be about 2,000.—The International Commission are still pursuing the inquiry into the North Sea incident, and on Monday the British and Russian Governments presented their respective conclusions, which are, of course, very conflicting. The Russian Government, while denying that responsibility for the outrage can possibly rest on Admiral Rozdestvensky or any of his colleagues, deplored that there should have been innocent victims of the accident, declared that there is no intention to evade material compensation, and suggested that the fixing of such compensation should be submitted to a tribunal to be chosen from The Hague Arbitration Court.

### Death of a Celebrated German Painter.

On Thursday last week Adolf Menzel died at his residence in Berlin from the effects of influenza. Born at Breslau in 1815 he was confessedly the greatest German painter of the nineteenth century. Two years ago some of his drawings just completed were exhibited in London and, perhaps, the most remarkable fact of his distinguished career was that between his 15th and his 90th year he laboured with ceaseless persistency and success. At the start he was physically insignificant; he was hampered by every disadvantage, without means and without training, and though he made some sensation in artistic circles in 1837 when he produced "The Chess Players," it was not until 1839, when he commenced his series of 400 illustrations for the official history of Frederick the Great, that he began to be famous. Having educated himself as a painter, he was entrusted in 1861 by King William of Prussia to paint a picture of his coronation at Konigsberg, and from that date his reputation was made. His pictures dealing with the Franco-Prussian war were among his many striking successes. A State funeral took place on Tuesday, and a guard of honour selected from the Berlin garrison by Imperial command, joined in the ceremony, the whole city being in mourning.

### The Russian Giant.

The "Giant Machnow," who has just been added to the attractions of the Hippodrome, is said to be the tallest man in the world. His height is 9 feet 3 inches, and his hands measure 2 feet from the wrist to the tip of the middle finger. In weight he is 4 lb. under 26 stone. Machnow is a native of Charkow, in Asiatic Russia, and is only in his 23rd year. He is in no sense a freak, and, apart from his size, has all the appearance of an ordinary man. His proportions are symmetrical, and, attired in a well-fitting long brown braided coat and Russian boots, with a white Persian lamb's-wool cap, he is quite a picturesque figure.

### Notes and Queries.

**REGULATIONS.**

**The Editor is always willing to answer in this column, without any fee, all reasonable questions, as soon as possible.**

**But the following rules must be carefully observed :—**

1. **Every communication must be accompanied by the name and address of the writer.**
2. **The question must always bear upon nursing, directly or indirectly.**

**If an answer is required by letter a fee of half-a-crown must be enclosed with the note containing the inquiry.**

*School Board Nurses.*

(147) Will you kindly tell me where to obtain particulars of the School Board nurses for London or Birmingham ?—*C. F. S.*

Write to the Principal Clerk, School Board for London, Victoria Embankment, S.W.

*Malta.*

(148) Can you tell me how or where I should apply for particulars for nursing in Malta, either for midwifery or maternity ?—*J. S.*

The hon. secretary of the Colonial Nursing Association, Imperial Institute, S.W., might help you with information. You might also write to the secretary of the Soldiers' and Sailors' Families Association, 23 Queen Anne's Gate, Westminster, S.W. At Malta two hospitals take paying patients, and possibly receive maternity cases. They are the Central Civil Hospital, Valetta, and the Santo Spirito Hospital. Write to the matrons.

*Nurses' Total Abstinence Association.*

(149) Will you kindly tell me if there is a Nurses' Total Abstinence Association, and, if so, to whom I should apply for information about it ?—*E. M. C.*

Write to Miss Dillon, Oakley Street, Chelsea, S.W.

*Sanitary Institute in Manchester.*

(150) Will you please tell me if it is possible to obtain the certificate of the Sanitary Institute in Manchester ? How should I set about it, and are there any books on the subject that you would advise me to read ?—*C. W.*

Write to Dr. Niven, Officer of Health, Manchester.

*Name of a Nurses Co-operation.*

(151) Will you kindly tell me whether there is a nurses' co-operation in London called the "Oxford," and, if so, where ?—*J. E. S.*

We know of no such co-operation, but it may be one of the multitudes of private associations of which there is no authentic list published.

*Regimental Midwives.*

(152) Can you tell me if there are such appointments as regimental midwives, and, if so, where to apply ?

Write to the matrons of the Woolwich Hospital for Soldiers' Wives and Children, and the Portsmouth Hospital for Soldiers' Wives and Children. Also to the secretary of the Soldiers' and Sailors' Families Association, 23 Queen Anne's Gate, Westminster, S.W. This association has a nursing branch which supplies qualified district nurses to attend the families of soldiers and sailors in large garrison and seaport towns.

*Massage.*

(153) Can you please tell me of any institutions for massage in or near London ?—*F. J. S.*

If you desire to be trained you had better apply to the National Hospital for the Paralysed and Epileptic, Queen Square, W.C.

*Fund for Helping Invalid Nurses.*

(154) Will you kindly tell me if you know of any fund or society for helping an invalid nurse ? . . . When she was younger she might have joined the Pension Fund, but she had an invalid mother dependent upon her.—*M. B.*

Write to the Hon. Sec. of THE HOSPITAL Convalescent Fund. As the nurse in question is so ill she should be eligible for some home or hospital for chronic and incurable diseases. There is a full list in "Burdett's Hospitals and Charities," which is published by the Scientific Press, 28 Southampton Street, Strand, London, W.C.

**Handbooks for Nurses.**

| | Post Free. |
|---|---|
| "The Nursing Profession: How and Where to Train" ... | 2s. 4d. |
| "The Nurses' Pronouncing Dictionary" ... ... ... | 2s. 0d. |
| "Nursing: its Theory and Practice" (Lewis) ... ... | 3s. 6d. |
| "Surgical Bandaging and Dressings" ... ... ... | 2s. 0d. |
| "A Complete Handbook of Midwifery" (Watson) ... | 6s. 4d. |

**Of all booksellers or of The Scientific Press, Limited 28 & 29 Southampton Street, Strand, London, W.C.**

### For Reading to the Sick.

DIVINE WISDOM.

GLORIOUS it is to wear the crown
Of a deserved and pure success :—
He who knows how to fail has won
A Crown whose lustre is not less.

Great may he be who can command
And rule with just and tender sway ;
Yet is diviner wisdom taught
Better by him who can obey.

Blessèd are those who die for God,
And earn the Martyr's crown of light—
Yet he who lives for God may be
A greater Conqueror in His sight.

*A. A. Proctor.*

A fourth mark of the spiritual life, a fourth fruit of the Spirit, is Long-suffering. And Long-suffering is perhaps that power which enables us to suffer on, which will not let us become ruffled, or put back, or paralysed, or overwhelmed by difficulties as they come upon us. For we must all suffer—there is no question about that—only suffering affects people in different ways ; some are broken and prostrated, while others are braced, chastened, and purified by it. And therefore it will be well, it may be, to give the word Long-suffering a very full meaning, to look upon it as the power of bearing with suffering, assimilating it, utilising it, instead of being irritated, fretted, and put out by it.

We must learn to bear long with ourselves. If we think of some time of special effort—such as last Lent, it may be—or some moment of earnest resolution, what fruit have we to show ? Oh ! so little. But still, just a little ; and, after all, a little is better than no fruit at all. May it not be that, as in a great workshop we may see strewn about half-finished pieces of work, impaired by some flaw, or vitiated by some artistic defect, which have been thrown away and discarded, and then some master workman comes round and picks them up, and says that they can be worked again into something else, for the metal is good—so, may it not be that if by God's great mercy we reach heaven at last, we shall find some of our failures there, in the special house, made up again into something else ? For honest good work is never lost ; and He Who gathers up the fragments cherishes our broken efforts and half-formed resolutions.

There is as much done by suffering as by action in the world. As has been well said, "There are no little things in the world while God concerns Himself with all" ; or, again, "Behind the veil there may be grades of greatness, but nothing insignificant." Above all, I must keep close to God ; all patient endurance is a gift of the Holy Spirit. My nature is to be impatient, hasty, fretful, weary, rebellious ; but long-suffering is above Nature—it is a fruit of the Spirit.

*Canon Newbolt.*

If thou art stricken for His blessed sake
Who bare for thee the scourge's cruel sting,
Rejoice, thy Lord hath called thee to partake
Of His own baptism of suffering ;
Low in that olive-shade He bowed for thee ;
Bear on—remembering Gethsemane.

